# Quality assessment of maize tortillas produced from landraces and high yield hybrids and varieties

**DOI:** 10.3389/fnut.2023.1105619

**Published:** 2023-02-09

**Authors:** Beatriz A. Acosta-Estrada, Sergio O. Serna-Saldívar, Cristina Chuck-Hernández

**Affiliations:** ^1^Tecnológico de Monterrey, School of Engineering and Sciences, Monterrey, Mexico; ^2^Tecnológico de Monterrey, The Institute for Obesity Research, Monterrey, Mexico

**Keywords:** Landraces, maize (Zea mays L.), dry masa flour, tortillas, quality assessment

## Abstract

**Introduction:**

Different analyses of the profiles of tortillas have been made using the traditional method, whether from landraces or hybrids versus those made with dry masa flour in which significant variability (*p* < 0.05) is reported in favor or against each type of tortilla which may be due to various factors such as the type of maize or the processing methods.

**Methods:**

Twenty-two samples including hybrids, hybrid mixtures, varieties, landraces and dry masa flours were processed to masa and tortilla under similar and controlled conditions and tortilla quality evaluated. In total, 70 characteristics were analyzed as physicochemical properties of the maize (e.g., hectoliter weight and dimensions), processability characteristics, masa characteristics [e.g. viscoamylographic parameters (RVA)], and quality parameters of tortillas (e.g., sensory performance, color and texture).

**Results and discussión:**

The studied materials presented variability among genotypes, especially within landraces. The physical and chemical properties of corn affected the processability and quality characteristics of tortillas (sensory and composition), and it was found that high producing hybrids and varieties (*p* < 0.05) were better and more consistent in all stages of processing. Forty percent of the landraces yielded masa with poor machinability.

**Conclusion:**

Landraces averaged 1.27 percentage points more protein (*p* < 0.05) than other analyzed samples and they comparatively yielded tortillas with lower extensibility (12.34%) compared to counterparts produced from hybrids and varieties. This work provides valuable information on how the chemical and physical characteristics of different types of maize genotypes affect the nixtamalization process and the quality of tortillas to provide more elements in the selection of the most appropriate genotypes for tortilla production.

## 1. Introduction

Corn (*Zea mays* L.) is the most important cereal crop in terms of total production. The global annual production reached 1,162 million tons, with the United States, China and Brazil as the main producing countries. Mexico is considered the cradle of this important crop and the place with higher genetic versatility and the origin of numerous races that have been used for development of open pollinated varieties and high producing hybrids. In 2020, the Mexican production of maize was about 27.4 million tons (1) and this output is mainly used to produce table or soft tortillas. It is estimated that about 96.5% of the tortillas are manufactured from varieties and hybrids (86% white and 14% yellow) and only 3.5% from landraces (2).

In terms of tortilla production, the preferred maize contains a regular endosperm which contains starch with about 25% amylose and 75% amylopectin and tortillas with different colors are produced from white, yellow, red, blue and purple corns. The use of white dent maize is growing because is the preferred for tortilla and related snacks production. Yellow colored tortillas can be produced from yellow maize rich in carotenoids or masa from white maize that is supplemented with extra lime. Colored genotypes, especially blue kernels, contain significant amounts of anthocyanins which exert potent antioxidant properties (3).

The chemical and physical properties of the maize kernels play the most critical role in the manufacture of nixtamalized products such as tortillas because they affect the product’s quality and strongly influence the processing parameters. Unfortunately, few scientific studies correlate maize characterization tests with the lime cooking or nixtamalization process and product variables, especially those related to tortilla quality that emphasizes in hybrids, varieties and landraces. Genotypes tailored-bred for nixtamalized products have improved processing efficiency. They possess an intermediate endosperm texture, medium to large kernels and comparatively higher test weight values, true density, and thousand-kernel weight (4). In contrast to the preferred genotypes for nixtamalization, most of the landraces have a soft-textured endosperms. About hundreds of landraces of maize are currently grown in Mexico; these have essential variations in their grain qualities, with physical and chemical differences among landraces (5, 6).

Dry masa flours (DMF) are obtained by grinding nixtamal with less moisture content than nixtamal used in fresh masa, which prevents the release of starch granules from the protein matrix (7), so the particles of the masa produced traditionally are different from counterparts associated to the DMF. Nevertheless, the amount of starch and protein of tortillas produced by the two sources are similar. In Mexico, 30% of the tortillas are produced from dry masa flour and the other 70% directly from nixtamalized grains (8). The main advantage of using DMF is the saving of processing time and equipment related to the bottleneck steps of lime-cooking and steeping (which generally last 8 to 16 h). The drying process during the production of DMF is a critical factor because it affects the degree of starch gelatinization which impacts water retention capacity and masa properties (7, 9). Among the main physicochemical properties related to the functionality of DMF are the particle size distribution, pH, water absorption capacity and masa rheology (7), in addition of being formulated to develop the flexibility and cohesivity of tortillas (10).

The acceptability of the final product processed by traditional nixtamalization depends on the structural changes generated in the grain, which result in rheological, functional and textural properties. Starch gelatinization is one of the structural changes most affected by factors such as cooking times and milling operations (11).

In the scientific literature, different analyses of the profiles of texture, composition and nutritional profiles tortillas were made using the traditional, industrial, or ecological method whether from landraces or hybrids, vs. those made with dry masa flour (5–7, 9, 11). In general terms, there is an agreement in the reported moisture contents. However, for other characteristics, significant variability is reported in favor or against each type of tortilla which may be due to various factors such as the type of maize or the processing methods, which generates a lot of confusion and lends itself to being whether you prefer a specific genotype. Therefore, research is needed in which all materials are processed to masa and tortilla under similar and controlled conditions in the laboratory, thus eliminating the acquisition of different methods and procedures in the thousands of mills and tortilla factories in Mexico. To our knowledge, this is the first study to be carried out under controlled conditions and with so many genotypes of corn.

This research related critical important grain properties to the processing and quality of tortillas and focused on differences between high yield hybrids and varieties, landraces, and dry masa flours. In addition, it is attempted to explain the reason why some commercial tortilla processors prefer to employ mixtures between high yield hybrids.

## 2. Materials and methods

### 2.1. Chemicals and reagents

Calcium hydroxide, ethanol, sodium hydroxide, chlorhydric acid, hexane, methanol, ethyl acetate, sodium phosphate, cupric sulfate, sulfuric acid, sodium nitrate solution and sodium carbonate were purchased from DEQ (Desarrollo de Especialidades Químicas, Mexico) and all reagents were of analytical grade. Folin–Ciocalteu reagent 2 N, and ferulic acid standard were purchased from Sigma-Aldrich (St. Louis, MO).

### 2.2. Corn genotypes

Twelve corn samples were collected in 2021, among which were seven high producing hybrids and varieties (HPHV) [hybrids: Corteva P4279W (provenance Campeche from spring–summer 2020 crop cycle), Corteva P4028W (provenance Chiapas from spring–summer 2020 crop cycle), Bayer DEKALB 2037 (provenance Bajio from spring–summer 2020 crop cycle), Bayer Antilope/Berrendo (provenance Jalisco from spring–summer 2020 crop cycle), and Bayer DEKALB 4050 (provenance Sinaloa from autumn-winter 2020), and varieties: INIFAP Quality Protein Maize (provenance experimental sites of the National Institute of Research in Forestry, Agriculture and Livestock from spring–summer 2020 crop cycle), and INIFAP High oil corn (provenance experimental sites of the National Institute of Research in Forestry, Agriculture and Livestock from spring–summer 2020 crop cycle)] and five landraces [Olotillo (provenance Oaxaca from spring–summer 2020 crop cycle), Serrano Mixe (provenance Oaxaca from spring–summer 2020 crop cycle), Chalqueño (provenance Puebla from spring–summer 2020 crop cycle), Native Texhuaca (provenance Estado de México from spring–summer 2020 crop cycle), and Native Blue (provenance Estado de México from spring–summer 2020 crop cycle)]. Six hybrid maize mixtures that are commonly used in different regions of Mexico [Nuevo León (provenance Sinaloa and Nuevo León from 2020 crop cycle), Estado de México (provenance Bajio and Jalisco from 2020 crop cycle), Bajío (provenance Sinaloa and Bajio from 2020 crop cycle), Jalisco (provenance Jalisco from 2019 and 2020 crop cycles), Veracruz (provenance Sinaloa from 2020 crop cycle), and Chiapas (provenance Sinaloa from 2020 crop cycle)] were also collected as well as six industrially produced dry masa flours (DMF) from these blends (moisture contents between 8.34–10.52%). The corn genotypes and flours were purchased from a national distributor based in Monterrey, Nuevo León. The samples were fumigated with aluminum phosphide (phostoxin tablets) to prevent insects and stored at 23°C until use.

### 2.3. Physical grain properties

Grain test weight was determined using the Winchester Bushel tester (model 60,607, Seedburo Equipment Company, Chicago, IL) according to AACC international (12) method 55–10.01. Thousand-kernel weight was quantified by weighing 100 randomly selected grains and multiplying its weight by 10. To determine the flotation index, 600 ml of sodium nitrate solution (41% w/v) at 23°C (density 1.25 g/ml) was used to quantify the floating grains. One hundred randomly selected grains were manually stirred (1 min) into the solution. In order to assess the index, the floating grains were separated and counted (13).

The percentage (ratio) of hard/vitreous endosperm was calculated with image analysis, through a scanner. The grains were placed on the scanner, the image was taken, and the calculations were made with the Winseedle software (Regent Instruments Inc.) (14). Grain dimensions (length and width in mm) were measured using an electronic vernier. The moisture content of samples was determined according to the method approved by the AACC 44–15.02.

The pericarp, endosperm, germ, and tip cap were manually dissected after soaking 10 kernels for 10 min in 100 ml of water. After draining off the water, the kernels were manually dissected into pericarp, endosperm, germ, and tip and dried for 48 h in an oven (VWR Forced Air Oven 1321F, Radnor, PA) at 105°C. Dry fractions were weighed (Mettler Toledo XS64, Columbus, OH) to calculate relative percentages.

### 2.4. Transformation to nixtamal, masa and tortilla

#### 2.4.1. Nixtamal

The optimal cooking time (OCT) and dry matter loss (DML) of the maize samples were determined according to the nylon bag procedure described by Serna-Saldivar et al. (15). Linear regression equations were calculated to predict optimal cooking and DML incurred during the different cooking times. The OCT was considered sufficient to increase the moisture of the nixtamal to 50% after 15 h of steeping.

For grain samples, batches of 3 kg were nixtamalized. Briefly, 3,000 g of clean corn and 30 g of lime (Quimex 97, Grupo Calidra, Mexico) were placed in a cooking vessel with water (9,000 ml) at 95 ± 2°C with stirring at 20 rpm for OCT and then left for 15 h steeping without stirring. After steeping, the nixtamal samples were washed by hand 3 times for 3 min with 3.5 l of water. The nejayote and wash waters were separated from the nixtamal using steel colanders. The cleaned and washed nixtamal samples were placed in a plastic bag to prevent moisture loss before masa preparation. Samples were characterized by their moisture content, according to the method approved by the AACC 44–15.02.

The remaining attached pericarp was determined by the May-Gruenwald stain test. A sample of 10 kernels was used. Grains were immersed using a perforated wire basket in May-Gruenwald stain (0.5 g eosin Y and 0.5 g methylene blue in 400 ml methanol) for 15 s. Subsequently, the grains were immersed for washing excess dye in 2 consecutive beakers with methanol for 10 s (13). The percentage of remaining pericarp was subjectively analyzed by measuring the proportion of pericarp (stained blue) remaining in the nixtamal and expressed as % of pericarp in the nixtamal.

#### 2.4.2. Masa

Masa was produced from 4,500 g of nixtamal which was ground in a stone mill adjusted to yield a fine masa suitable for tortilla production. Water was added during grinding to increase the masa moisture to 57% and to avoid excessive temperature due to the friction between the grinding stones. Dry Masa Flour (DMF) samples (2000 g) were reconstituted into masa kneading the flour with water at 80 rpm. The water absorption and kneading time was calculated with the help of Mixolab (Mixolab 2, Chopin Technologies) following the protocol for nixtamalized maize flour (16) where the optimal water absorption was the necessary to achieve a torque (C1) of 1 Nm. Part of the sample was placed in plastic bags and the remaining was lyophilized for later determinations.

#### 2.4.3. Tortilla

For tortilla production, masa samples were continuously laminated and formed into circular pieces with a commercial sheeter/former (Model V-25 comal en banda, Grupo Villamex, Mexico). The weight of the round pieces (12 cm in diameter) were set at 21 g. The masa disks were baked for 60 s into soft tortillas in a three tier gas-fired oven, at an average temperature of 148°C. Masa for tortillas was blended with 0.25% sodium propionate, 0.3% fumaric acid and 0.1% sorbic acid in order to prevent microbial contamination for texture and rollability determinations. Part of the sample was placed in plastic bags and another part was lyophilized for later determinations.

### 2.5. Masa quality assessment

The assessment of the quality of fresh nixtamalized maize masa performed with Mixolab (Mixolab 2, Chopin Tecnologías) analyses as reported by Espinosa-Ramírez et al. (17). For pH measurement in fresh masa, a 10 g sample was placed in a blender with 100 ml of distilled and blended at high speed for 2 min The contents were allowed to settle for 5 min. The pH was measured with a potentiometer previously calibrated with three buffer standards (pH 4, 7 and 10).

A sample of freeze-dried masa was carefully disrupted before the assessment of granulometry distribution. The particle size distribution of mass d(0.1) μm, d(0.5) μm and d(0.9) μm was analyzed, representing the maximum diameter of 10, 50, 90 and 98% of the particles, respectively (Supplementary Table S1). For this, a particle analyzer based on laser diffraction (Mastersizer 2000, Malvern, Instruments, United Kingdom) equipped with a Scirocco unit (dry powder unit) was used. The equipment has a measurement range of 0.02–2000 μm. The measurement parameters were set in accordance with the ISO 13320-1 standard. The Mie theory was applied considering a refraction index of 1.52 and an absorption index of 0.1.

The pasting profiles of dry masa flours previously ground with the Udy Mill (Cyclone Sample Mill no. 3010–014, Udy corporation, Fort Collins, CO; No. 80 US sieve) were determined by a Rapid Visco Analyzer (RVA; Perten Instruments, Australia). A concentration of 3 g sample corrected for a basis of 14% moisture/25 ml distilled water was used. The dispersion was heated to 50°C for 1 min with stirring at 190 rpm, followed by a decrease on stirring speed to 160 rpm and a temperature increase up to 95°C withing 3.5 min, held at 95°C for 5 min, cooled to 50°C within 3.5 min and then held at 50°C for 4 min. Analysis was performed in duplicate. Data was analyzed by Termocline Software TCW3 3.15.3.347 and parameters peak 1, through 1, breakdown, final viscosity, setback, peak time and pasting temperature were obtained.

### 2.6. Tortilla quality assessment

The color of fresh tortillas was measured with a colorimeter (Konica, Minolta, Japan). Color values L* (lightness), a* (red-green), and b* (yellow-blue) were determined. Rollability determinations (*n* = 5) of fresh (day-0) and 1-day, 3-day, and 7-day stored tortillas were evaluated. Tortillas were wrapped around a 1 cm diameter acrylic bar. The degree of breakage was determined using a subjective scale from 1 to 5. A score of 1 represented a 100% broken tortilla or torn tortilla; a score of 3 indicated a broken tortilla or 50% break in the tortilla structure whereas a score of 5 indicated an unbroken tortilla considered the best rolling ability.

Texture (*n* = 5) of fresh (day-0) and 1, 3, and 7-day stored tortillas was evaluated using the TVT 6700 texture analyzer (Perten Instruments, Australia), equipped with the Tortilla Burst Rig (HDP/TPB) platform and a spherical probe (25 mm). Tortilla samples were stretched at a rate of 1 mm/s to determine breaking strength and extensibility (maximum extension before breaking).

Sensory evaluation tests were carried out with 10 trained panelists with an age range of 25 to 41 years and an average of 30.6 years. 40% of the participants identified themselves as men and 60% as women. Each panelist simultaneously received 3 to 5 coded reheated samples along with a glass of water and a ballot, and was asked to rate color, texture, taste, odor, and general acceptability on a 9-point hedonic scale. Testing was conducted with one-day old control (commercial sample) and 1 day old test tortillas.

### 2.7. Tortilla proximate composition and total phenolic content

Samples were characterized for moisture content, crude protein, crude fat, and ash according to AACC approved methods 44–15.02, 46–13.01, 30–10.01, and 08–01.01, respectively (12). Total carbohydrates were calculated by the difference: 100 – protein – fat – ash as dry basis. Total starch was determined by the Megazyme (Wicklow, Ireland) Total Starch Assay Kit (AA/AMG) K-TSTA-100A.

For the determination of phenolic compounds, 1 g of dry ground tortilla sample was mixed with 20 ml of 80% ethanol for 10 min in a shaker (Incubator with orbital shaker, Mrc Laboratories, Israel) at 250 rpm and 25°C and then centrifuged (Thermofisher Scientific SL 16R, Waltham, MA) at 3000 g (10 min and 4°C). The supernatant was recovered and stored at −80°C until use. The bound phenolic compounds were extracted from the resulting pellet. Alkaline hydrolysis (10 ml NaOH 2 M) was performed for 1 h, the samples were then acidified with 2 M HCl to pH 2. The acidified samples were extracted five times with 10 ml of ethyl acetate and the fractions were evaporated to dryness. The bound phenolics were resuspended in 50% methanol and stored at −80°C until use (18).

Total phenolics were determined by the Folin–Ciocalteu colorimetric method. Twenty ml of the appropriate sample dilutions were oxidized with 100 ml of 10% v/v Folin–Ciocalteu reagent in distilled water and the reaction neutralized with 80 ml of 7.5% w/v sodium carbonate in water. After incubation (1.5 h at 37°C in the absence of light), the absorbance at 765 nm was measured using a microplate reader (Synergy^TM^ HT Multi-Detection, BioTek, Inc., Winooski, VT). Ferulic acid was used as a standard and the total phenolic content was expressed in mg of ferulic acid equivalents (FAE / 100 g tortilla dry base) (18).

For the differential scanning calorimetry (DSC) analysis (Supplementary Table S2), 3 mg of ground tortilla was placed in semi-hermetic anodized aluminum capsules (Perkin Elmer, B02190062, United States), hydrated with the appropriate amount of distilled water (3 volumes, based on the total weight of the sample) and containers were carefully sealed containers. Once hydrated, the samples were kept for 24 h at room temperature (25°C) and subsequently heated from 30 to 90°C at an incremental rate of 10°C/min in a Diamond DSC apparatus (Perkin Elmer, Nortfolk, VA, United States) calibrated with an aluminum reference cell before the experimental measurements were made. An empty capsule was used as reference for each determination (19).

### 2.8. Statistical analysis

Each experiment was performed in triplicate unless otherwise specified, and data was reported as mean ± standard deviation. Results were subjected to analysis of variance and differences among means were compared by Tukey tests at *p* < 0.05 in Minitab version 19.2020.1 (SAS Institute Inc. Cary, NC). Pearson correlations were performed using the Spearman method (*p* < 0.05) to assess the monotonic relationship between variables.

## 3. Results and discussion

### 3.1. Physical properties

Ideal characteristics preferred for lime cooking are generally found in dent corn that have a medium endosperm texture (translucency), medium to large kernels (1,000 kernel weight of 320 g), a rounded crown, and a shallow, wrinkle-free dent (4). The corns studied herein had a weight of 1,000 grains between 210.13 and 433.57 g (Table 1). As a whole, HPHV and landraces have an average 1,000 kernel weight value close to the ideal, but the landraces grains show great variability, having values that ranged from 210 g to 433 g, while the range of the hybrids was more consistent (256 to 343 g), where mixtures can be made with them to obtain one with characteristics similar to the ideal one.

**Table 1:** Physical properties of landraces, commercial maize and mixtures of hybrid maize varieties.

Sample		Test weight				Dimensions (mm)								Flotation index				Vitreous endosperm				1 K kernel weight				Moisture			
		(Kg/hL)				Length (mm)				Width (mm)				(%)				(%)				(g)				(%)			
H	Corteva P4279W	78.07	±	0.21	b	11.65	±	0.80	b	8.94	±	0.97	ab	13.3	±	5.7	efg	97.0	±	0.3	b	264.77	±	10.43	g	14.80	±	0.001	b
H	Corteva P4028W	78.30	±	0.08	b	11.63	±	0.88	b	8.77	±	0.75	ab	7.7	±	0.5	fg	97.0	±	0.7	b	256.87	±	1.89	gh	14.07	±	0.000	cde
L	Olotillo	73.83	±	0.41	de	13.60	±	0.89	ab	6.86	±	0.71	b	23.7	±	1.9	de	90.0	±	0.2	d	231.37	±	4.22	hi	13.87	±	0.001	de
L	Serrano Mixe	79.83	±	0.25	a	11.00	±	0.91	b	8.33	±	0.87	ab	8.7	±	1.7	fg	100.0	±	0.3	a	210.13	±	3.21	i	13.90	±	0.001	cde
L	Chalqueño	68.73	±	0.25	g	15.52	±	1.06	a	9.68	±	0.99	ab	73.3	±	4.6	b	31.0	±	0.7	j	381.87	±	12.66	b	13.97	±	0.001	cde
H	Bayer DEKALB 2037	74.13	±	0.31	de	12.62	±	0.65	ab	9.11	±	0.64	ab	8.3	±	2.6	fg	83.0	±	0.0	f	321.63	±	12.56	de	12.67	±	0.001	gh
L	Native Texhuaca	72.07	±	0.26	f	14.46	±	2.52	ab	8.79	±	1.57	ab	40.0	±	0.8	c	74.0	±	0.8	h	380.87	±	20.94	b	12.93	±	0.000	fg
H	Bayer Antilope/Berrendo	75.77	±	0.17	c	12.32	±	0.60	ab	9.03	±	0.69	ab	25.7	±	3.1	d	97.0	±	0.7	b	283.63	±	9.16	fg	13.20	±	0.001	f
L	Native Blue	67.07	±	0.45	h	14.31	±	1.04	ab	9.13	±	0.86	ab	88.7	±	1.7	a	0.0	±	0.0	k	433.57	±	6.13	a	14.70	±	0.000	b
H	Bayer DEKALB 4050	73.03	±	0.39	ef	12.91	±	0.68	ab	9.05	±	0.91	ab	21.7	±	4.1	de	87.0	±	0.8	e	343.87	±	4.78	cd	14.27	±	0.001	c
V	INIFAP Quality Protein Maize	79.70	±	0.14	a	11.56	±	0.48	ab	10.09	±	0.53	a	4.0	±	0.5	g	83.5	±	0.4	f	328.70	±	4.91	cde	12.46	±	0.002	h
V	INIFAP High oil corn	77.87	±	0.09	b	11.68	±	0.62	ab	8.56	±	0.70	ab	4.0	±	0.8	g	99.0	±	0.0	a	286.90	±	9.36	fg	12.93	±	0.000	fg
M	Nuevo León	75.53	±	0.40	c	12.49	±	0.72	ab	8.84	±	0.68	ab	10.3	±	3.7	fg	83.0	±	0.5	f	359.60	±	5.03	bc	14.07	±	0.000	cde
M	Estado de México	75.43	±	0.21	c	12.51	±	1.01	ab	8.67	±	0.75	ab	14.0	±	2.2	efg	83.0	±	0.5	f	311.93	±	8.89	def	13.90	±	0.000	cde
M	Bajío	75.00	±	0.08	cd	12.70	±	0.91	ab	8.82	±	0.74	ab	17.7	±	0.5	def	93.0	±	0.5	c	304.57	±	4.18	ef	14.13	±	0.001	cde
M	Jalisco	73.23	±	0.45	ef	12.04	±	0.84	ab	8.80	±	0.81	ab	10.3	±	2.6	fg	77.0	±	0.1	g	358.80	±	6.01	bc	13.77	±	0.002	e
M	Veracruz	75.90	±	0.16	c	12.56	±	0.88	b	8.78	±	0.80	ab	17.0	±	4.3	def	77.0	±	0.2	g	360.50	±	1.88	bc	14.20	±	0.001	cd
M	Chiapas	76.07	±	0.68	c	12.56	±	0.88	b	9.00	±	0.76	ab	25.0	±	1.6	d	57.0	±	0.5	i	270.20	±	6.01	g	15.27	±	0.001	a
High producing hybrids and varieties		76.70	±	2.26	A	12.05	±	0.51	B	9.08	±	0.45	A	12.1	±	7.9	B	91.9	±	6.6	A	298.05	±	31.01	A	13.48	±	0.827	A
Landraces		72.31	±	4.46	A	13.78	±	1.52	A	8.56	±	0.96	A	46.9	±	30.0	A	59.0	±	37.8	A	327.56	±	89.52	A	13.87	±	0.562	A
Hybrids mixtures		75.19	±	0.94	A	12.48	±	0.20	AB	8.82	±	0.10	A	15.7	±	5.0	B	78.3	±	10.9	A	327.60	±	34.52	A	14.22	±	0.489	A

H = Hybrid maize; V = Maize varieties; L = Landraces; M = Hybrid mixtures. Means with a different letter(s) within genotypes and groups of genotypes columns are statistically different (*p* < 0.05). All data was reported as mean ± standard deviation.

Likewise, the ideal maize genotypes for the industry have a test weight of 76–78 kg/hl, and true density of 1.3 g/cm^3^ (20). The grain mixtures were optimized to have a specific weight around 75 kg/hl, using hybrid grains with little variability (73.03–79.70 kg/hl) while the landraces (67–79.8 kg/hl) presented greater dispersion, therefore mixtures would have to be devised for landrace’s optimal use.

In general, higher variability was observed in the physical characteristics of landraces, obtaining the highest and lowest values of all the genotypes evaluated, since the hybrids are selected and bred to have certain characteristics.

Although the landraces had greater variability, as a group they were not statistically different, only in the characteristics of flotation index and size (length) of the kernels (Table 1). Interestingly, flotation index and size (length) of the kernel correlated with each other (ρ = 0.788). The landraces had an endosperm of intermediate hardness, while the hybrids and mixtures had a harder endosperm texture. Blue maize is a floury or soft endosperm type (21) and the only sample with 0% vitreous endosperm (Table 1). QPM had intermediate endosperm texture because they maintained the cysteine-rich gamma-zeins (20) and had similar value of vitreous endosperm (83%) (Table 1) to the mixtures. Kernel hardness was correlated with performance parameters in the grain-nixtamal transformation process, such as dry matter loss (ρ = −0.556), which affects nixtamal yield, as well as other physical parameters [hectoliter weight [(ρ = −0.687), and vitreous endosperm (−0.557)]. The kernels of landraces were 14% longer than the hybrid genotypes. The length of the grains correlated with the 1,000 kernel weights (ρ = 0.557), and vitreous endosperm (ρ = −0.622), among others. The main criteria used to select maize for tortilla production relates to physical kernel parameters as they influence functionality and cooking parameters (20).

### 3.2. Nixtamal quality assessment

Maize is usually batch cooked by mixing with 2.5–3.0 parts water and 1% food grade lime based on kernel weight, depending on the weight of the kernel. Corn cooks evenly at temperatures above 68–70°C (the average starch gelatinization temperature of this cereal). The cooking process is generally divided into three steps: rise time, maintenance at the maximum programmed temperature and temperature drop-steeping (13). The optimal cooking time of the maize genotypes ranged from −23 min (the corn was placed 23 min after turning off the heat source) to 22 min with the heat source (Table 2). These latter samples required longer cooking times to achieve the desired nixtamal moisture level.

**Table 2:** Transformation of grain to nixtamal, masa and tortilla; process conditions and product characteristics.

Sample		Optimal cooking time (min)	Dry matter loss (%)	Kneading time (min)	Water absorption (%)	Subjective machinability	Remnant pericarp (%) *n* = 10				Moisture (%)												pH			
											Nixtamal				Masa				Tortilla				Masa			
H	Corteva P4279W	6.30	1.76	-	-	Good	15.10	±	16.67	c	49.01	±	0.48	abc	57.16	±	0.46	defg	39.18	±	0.51	h	8.65	±	0.21	a
H	Corteva P4028W	5.54	2.83	-	-	Good	6.80	±	5.75	c	48.36	±	0.45	abc	56.75	±	0.23	defgh	31.54	±	1.62	i	8.65	±	0.07	a
L	Olotillo	3.10	3.58	-	-	Bad	31.20	±	22.11	c	52.95	±	2.22	a	56.47	±	0.09	fgh	40.73	±	0.79	gh	8.05	±	0.07	cde
L	Serrano Mixe	8.00	3.53	-	-	Good	14.60	±	11.92	c	47.71	±	0.66	c	57.35	±	0.45	def	45.78	±	0.33	bcd	8.45	±	0.07	ab
L	Chalqueño	−14.93	1.65	-	-	Bad	19.90	±	24.79	c	52.63	±	2.22	ab	56.92	±	0.19	defgh	42.00	±	0.30	efgh	8.00	±	0.00	cdef
H	Bayer DEKALB 2037	0.20	2.81	-	-	Good	10.70	±	9.13	c	50.52	±	0.65	abc	56.62	±	0.06	efgh	43.50	±	1.26	defg	8.15	±	0.07	bcd
L	Native Texhuaca	15.12	3.02	-	-	Good	8.50	±	11.24	c	48.56	±	0.09	bc	55.65	±	0.12	hij	40.87	±	0.92	gh	8.20	±	0.00	bcd
H	Bayer Antilope/Berrendo	14.15	1.32	-	-	Good	18.90	±	12.63	c	51.63	±	0.85	abc	56.62	±	0.36	efgh	40.02	±	0.36	h	8.25	±	0.07	bc
L	Native Blue	−23.11	1.81	-	-	Bad	17.20	±	16.41	c	49.53	±	0.92	abc	55.89	±	0.11	ghij	44.21	±	0.30	bcdef	7.65	±	0.07	fgh
H	Bayer DEKALB 4050	−7.42	2.42	-	-	Good	14.50	±	10.45	c	47.88	±	0.56	c	55.49	±	0.61	hijk	39.16	±	0.00	gh	7.85	±	0.07	defg
V	INIFAP Quality Protein Maize	−8.13	4.31	-	-	Good	75.00	±	20.14	a	49.97	±	0.79	abc	57.92	±	0.22	de	46.38	±	0.38	bcd	7.45	±	0.07	h
V	INIFAP High oil corn	13.30	7.23	-	-	Good	17.80	±	11.26	c	48.95	±	0.39	abc	57.60	±	0.23	def	46.42	±	0.42	bcd	7.60	±	0.00	gh
M	Nuevo León	21.92	4.56	-	-	Bad	27.60	±	24.86	c	49.59	±	2.33	abc	57.50	±	0.98	def	44.95	±	0.08	bcdef	8.15	±	0.07	bcd
M	Estado de México	2.34	2.78	-	-	Good	13.60	±	13.70	c	48.19	±	0.77	c	56.71	±	0.15	defgh	44.57	±	1.39	bcdef	7.75	±	0.07	efgh
M	Bajío	−13.16	2.74	-	-	Bad	60.00	±	31.62	ab	48.93	±	1.11	abc	53.58	±	0.11	l	41.92	±	0.50	fgh	7.75	±	0.07	efgh
M	Jalisco	−4.29	3.02	-	-	Good	24.10	±	15.65	c	49.06	±	1.03	abc	54.18	±	0.26	kl	46.75	±	0.26	bcd	7.65	±	0.07	fgh
M	Veracruz	−3.30	3.74	-	-	Good	32.40	±	26.93	bc	47.37	±	0.17	c	54.55	±	0.08	jkl	45.54	±	0.54	bcdef	7.60	±	0.14	gh
M	Chiapas	−4.83	3.36	-	-	Good	14.40	±	10.96	c	48.72	±	1.21	c	55.23	±	0.55	ijk	44.63	±	0.49	bcde	8.05	±	0.21	cde
DMF	Nuevo León	-	-	1.17	134	Good	-				-				60.23	±	0.03	b	51.12	±	0.18	a	5.65	±	0.07	j
DMF	Estado de México	-	-	1.38	120	Good	-				-				58.27	±	0.05	cd	43.78	±	0.19	cdefg	6.55	±	0.07	i
DMF	Bajío	-	-	1.33	133	Good	-				-				60.91	±	0.03	ab	47.74	±	0.67	ab	6.60	±	0.00	i
DMF	Jalisco	-	-	1.90	136	Good	-				-				61.12	±	0.08	ab	46.91	±	2.15	bcd	5.85	±	0.07	j
DMF	Veracruz	-	-	1.92	135	Good	-				-				62.17	±	0.16	a	47.51	±	0.76	abc	5.65	±	0.07	j
DMF	Chiapas	-	-	1.65	128	Good	-				-				59.63	±	0.40	bc	46.26	±	0.88	bcd	6.80	±	0.00	i
High producing hybrids and varieties							22.69	±	21.70	A	49.47	±	1.21	A	56.88	±	0.73	B	40.89	±	4.80	C	8.09	±	0.44	A
Landraces							15.23	±	9.65	A	41.90	±	18.84	AB	47.05	±	21.05	B	35.60	±	16.02	BC	8.07	±	3.02	A
Hybrids mixtures							28.68	±	17.02	A	48.64	±	0.77	B	55.29	±	1.52	CD	44.73	±	1.59	AB	7.83	±	0.22	A
Dry masa flours							-				-				60.39	±	1.23	A	47.22	±	2.17	A	6.18	±	0.48	B

H = Hybrid maize; V = Maize varieties; L = Landraces; M = Hybrid mixtures; DMF = Dry masa flours. Means with a different letter(s) within genotypes and groups of genotypes columns are statistically different (*p* < 0.05). All data was reported as mean ± standard deviation.

During cooking and steeping, the kernel absorbs water and calcium mainly through the hilum and germ (4) and then the moisture is transported through the tube cells so the endosperm acquire moisture from the outer to the inner part by permeation (4). Normally the moisture content increases from 12–15% to 47–53% and nixtamal for table tortillas is cooked more extensively compared to nixtamal aimed for tortilla chips. Likewise, it was found that the optimal cooking time correlated positively with the pH of the masa (ρ = 0.561), and similarly the pH with the concentration of calcium in the tortillas (ρ = 0.746).

The nixtamalization conditions change all kernel components, from the pericarp to the endosperm. The pericarp should be easily removed during the first steps of nixtamalization for genotypes bred for nixtamalization products (22). Pericarp removal easiness is a heritable trait (20). Interestingly, the landraces contained 4.90–5.86% pericarp in contrast with high producing hybrids (4.50–5.19%), varieties (4.93–6.10%) and hybrid mixtures (4.51–5.10%; Table 3). Nevertheless, the remnant pericarp after nixtamalization was not statistically different among groups, but it was within hybrids and varieties, being the variety Quality Protein Maize (QPM) the one with the highest percentage of remnant pericarp (75%), followed by the hybrid Bajio mixture (60%) (Table 2). QPM is the result of breeding opaque-2 maize that was combined with modifier genes to improve hardness and agronomic performance (23) resulting in difficult to remove pericarp. Interestingly, the percentage of the remnant pericarp was correlated with the dietary fiber of the tortillas (ρ = 0.509).

**Table 3:** Anatomical parts of landraces, commercial maize and mixtures of hybrid maize varieties.

**Sample**		**Tip cap**				**Pericarp**				**Endosperm**				**Germ**			
		**(%)**				**(%)**				**(%)**				**(%)**			
H	Corteva P4279W	1.54	±	0.14	bc	5.19	±	0.34	bcdef	84.37	±	0.19	abc	8.89	±	0.12	abc
H	Corteva P4028W	1.63	±	0.17	bc	4.97	±	0.07	cdef	86.89	±	1.17	a	6.51	±	1.07	cd
L	Olotillo	2.72	±	0.11	a	5.86	±	0.10	ab	84.25	±	1.41	abc	7.17	±	1.40	bcd
L	Serrano Mixe	2.54	±	0.24	ab	5.49	±	0.26	abcde	82.39	±	1.92	c	9.58	±	1.43	a
L	Chalqueño	1.95	±	0.18	abc	5.56	±	0.65	abcd	86.95	±	1.10	a	5.54	±	0.46	d
H	Bayer DEKALB 2037	1.76	±	0.14	abc	4.53	±	0.09	f	84.86	±	0.31	abc	8.85	±	0.43	abc
L	Native Texhuaca	2.24	±	0.39	abc	5.77	±	0.38	abc	85.05	±	0.79	abc	6.94	±	0.12	cd
H	Bayer Antilope/Berrendo	2.18	±	0.12	abc	4.50	±	0.08	f	85.75	±	0.12	a	7.57	±	0.15	abcd
L	Native Blue	2.26	±	0.11	abc	4.90	±	0.04	def	85.15	±	0.61	ab	7.69	±	0.53	abcd
H	Bayer DEKALB 4050	1.47	±	0.11	c	4.50	±	0.06	f	86.01	±	0.14	a	8.03	±	0.09	abc
V	INIFAP Quality Protein Maize	2.55	±	0.79	ab	4.93	±	0.18	def	82.79	±	0.51	bc	9.72	±	0.80	a
V	INIFAP High oil corn	1.77	±	0.08	abc	6.10	±	0.11	a	82.79	±	0.29	bc	9.34	±	0.24	ab
M	Nuevo León	2.34	±	0.36	abc	5.10	±	0.11	bcdef	85.50	±	0.18	ab	7.06	±	0.46	cd
M	Estado de México	2.53	±	0.35	abc	4.86	±	0.16	def	84.50	±	0.01	abc	8.12	±	0.23	abc
M	Bajío	2.57	±	0.04	ab	4.51	±	0.15	f	84.99	±	0.45	abc	7.93	±	0.37	abcd
M	Jalisco	2.32	±	0.41	abc	4.77	±	0.11	def	85.51	±	0.41	ab	7.40	±	0.10	abcd
M	Veracruz	1.98	±	0.08	abc	4.61	±	0.07	ef	84.74	±	0.36	abc	8.67	±	0.33	abc
M	Chiapas	2.01	±	0.21	abc	4.82	±	0.21	ef	85.03	±	0.19	ab	8.14	±	0.25	abc
High producing hybrids and varieties		1.84	±	0.36	B	4.96	±	0.53	B	84.78	±	1.46	A	8.42	±	1.03	A
Landraces		2.34	±	0.27	A	5.52	±	0.34	A	84.76	±	1.48	A	7.39	±	1.31	A
Hybrids mixtures		2.29	±	0.23	A	4.78	±	0.19	B	85.05	±	0.37	A	7.89	±	0.52	A

H = Hybrid maize; V = Maize varieties; L = Landraces; M = Hybrid mixtures. Means with a different letter(s) within genotypes and groups of genotypes columns are statistically different (*p* < 0.05). All data was reported as mean ± standard deviation.

### 3.3. Masa quality assessment

Additionally, cooking with lime releases gums from the pericarp that impacts the viscosity, cohesion, and stickiness of masa and tortillas. Nixtamalization removes the pericarp producing masa with lower fiber content and more elastic (6). When processing masa into tortillas, it was subjectively classified as good and bad machinability (Table 2). The ideal masa should be cohesive and not sticky, and these properties are mainly controlled by the degree of starch gelatinization and particle size distribution of the masa. Masa with good machinability should form a curtain on the sheeting and forming rollers and the cut tortilla discs detach easily from the rollers, while those with poor machinability do not form a curtain due to the lack of cohesiveness or adhere firmly to the rollers due to stickiness.

The masa samples with poor machinability, among others, were the blue landrace (brittle and fragile) and the Chalqueño landrace (lacked cohesiveness, firm masa to touch). These samples had mostly floury endosperm (Table 1) and required the shortest cooking times (Table 2) of all the evaluated genotypes. The data indicated that the masa machinability correlated negatively with kernel length (ρ = −0.538).

The germ is also affected by the nixtamalization process, due to the diffusion of non-polar and polar lipids and the denaturation of proteins (albumins and globulins), favoring the cohesion of the components (due to the starch-protein-lipid interaction) (19). No differences were found among landraces, HPHV and hybrid mixtures in germ content. The variety QPM and the landrace Serrano Mixe were the ones with the highest germ content. When comparing Serrano Mixe with the Chalqueño landrace it was observed that the former contained 1.7 times more germ than the latter. In addition, the components of the modified germs affected the flavor and texture of the final product (10, 24). In particular, the oil content reduces water absorption and both polar and non-polar lipids improve tortilla texture by preventing starch retrogradation (19).

The different genotypes and their mixtures were processed under the same protocol, resulting in the same granulometry in the masa coming from nixtamal but not from dry masa flour (Supplementary Table S1). Typically, dry masa flours for soft tortillas are finer compared to counterparts employed for corn and tortilla chips (20).

Masa from dry masa flours contained 9.2 and 6.5% more moisture than those produced by hybrid mixtures and HPHV/landraces, respectively. This difference in water retention capacity has been previously reported as associated with the changes that occur in the starch chains during the drying of dry masa flours: production of short chains that retain more water molecules, in addition to the differences in the particles obtained after grinding, which for dry masa flours are carried out under low moisture conditions, which allows the production of particles with starch and proteins similar to those present in the maize kernel (7) and which affects their weighted average size (Table 1). Regarding yields in the total transformation, HPHV and landraces had the same yield (145%) and the mixtures of grains were superior with 10 percentage points above the HPHV and landraces (Supplementary Table S3). Total yield was positively correlated with dietary fiber in tortillas (ρ = 0.619).

**Figure 1:**
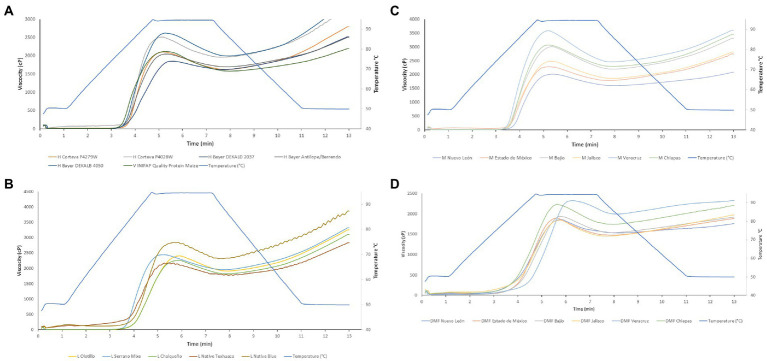
Rapid Visco Analyzer (RVA) curves of pasting properties of nixtamalized maize masa and flours from different genotypes. **(A)** High producing hybrids and varieties. **(B)** Landraces. **(C)** Hybrid mixtures. **(D)** Dry masa flours. Each curve was the average of two analyses. Different scales were used for each graph to differentiate values among samples. H = Hybrid maize; V = Maize varieties; M = Hybrid mixtures; DMF = Dry masa flours.

Total starch content also affected viscoelastic properties and masa consistency. The total starch content in the samples increased RVA breakdown (ρ = 0.517; Figure 1) and Mixolab retrogradation C5 (ρ = 0.525; Table 4), which impacted the texture of the tortilla by increasing hardness values.

**Table 4:** Mixolab parameters of nixtamalized maize masa and flours.

Samples		Initial consistency (C1), Nm				Peak torque during heating (C3), Nm				Cooking stability range (C3-C4), Nm				Retrogradation (C5), Nm				Stability time (tCs), min			
H	Corteva P4279W	0.94	±	0.08	def	1.08	±	0.00	cde	0.06	±	0.00	a	1.67	±	0.00	cde	1.60	±	0.28	f
H	Corteva P4028W	1.02	±	0.00	bcdef	1.10	±	0.00	cd	0.07	±	0.00	a	1.68	±	0.00	cde	1.50	±	0.00	f
L	Olotillo	1.21	±	0.25	abc	1.28	±	0.24	abcd	0.13	±	0.06	a	1.64	±	0.19	de	2.30	±	0.57	ef
L	Serrano Mixe	1.12	±	0.02	abcdef	1.37	±	0.20	abc	0.07	±	0.02	a	1.80	±	0.01	bcd	2.90	±	0.28	cdef
L	Chalqueño	1.01	±	0.03	bcdef	1.44	±	0.02	ab	0.14	±	0.07	a	1.87	±	0.15	bcd	3.10	±	0.42	bcdef
H	Bayer DEKALD 2037	1.29	±	0.05	a	1.28	±	0.11	abcd	0.11	±	0.03	a	1.87	±	0.01	bcd	1.65	±	0.21	f
L	Native Texhuaca	1.08	±	0.02	abcdef	1.38	±	0.04	abc	0.13	±	0.05	a	1.73	±	0.02	cde	1.50	±	0.14	f
H	Bayer Antilope/Berrendo	1.14	±	0.08	abcd	1.25	±	0.03	bcd	0.14	±	0.10	a	1.68	±	0.04	cde	1.50	±	0.14	f
L	Native Blue	0.95	±	0.03	cdef	1.50	±	0.00	ab	0.19	±	0.05	a	1.85	±	0.05	bcd	2.25	±	0.64	ef
H	Bayer DEKALD 4050	0.97	±	0.01	cdef	1.41	±	0.00	abc	0.16	±	0.00	a	2.02	±	0.01	bcd	1.85	±	0.49	f
V	INIFAP Quality Protein Maize	0.64	±	0.02	g	1.37	±	0.03	abc	0.19	±	0.10	a	1.92	±	0.22	bcd	2.30	±	0.14	ef
V	INIFAP High oil corn	0.87	±	0.02	efg	1.24	±	0.00	bcd	0.08	±	0.02	a	1.73	±	0.02	cde	3.60	±	0.71	abcde
M	Nuevo León	1.25	±	0.05	ab		BLD				BLD			2.00	±	0.13	bcd	1.65	±	0.07	f
M	Estado de México	0.85	±	0.04	fg	1.35	±	0.03	abc	0.10	±	0.05	a	1.90	±	0.00	bcd	2.90	±	0.14	cdef
M	Bajío	1.16	±	0.00	abcd	1.65	±	0.00	a	0.06	±	0.00	a	2.49	±	0.28	a	3.75	±	0.07	abcde
M	Jalisco	1.10	±	0.05	abcdef	1.53	±	0.01	ab	0.12	±	0.11	a	2.16	±	0.11	ab	4.30	±	1.13	abc
M	Veracruz	1.02	±	0.00	bcdef	1.47	±	0.02	ab	0.22	±	0.04	a	2.06	±	0.02	bc	2.65	±	0.07	def
M	Chiapas	1.14	±	0.05	abcde	1.42	±	0.03	ab	0.21	±	0.04	a	2.01	±	0.13	bcd	1.80	±	0.28	f
DMF	Nuevo León	0.99	±	0.04	bcdef	0.79	±	0.03	ef	0.07	±	0.02	a	1.10	±	0.01	f	4.05	±	0.21	abcd
DMF	Estado de México	0.95	±	0.07	def	0.97	±	0.03	def	0.10	±	0.01	a	1.34	±	0.02	ef	4.70	±	0.14	ab
DMF	Bajío	0.98	±	0.03	bcdef	0.77	±	0.01	ef	0.07	±	0.00	a	1.16	±	0.01	f	4.30	±	0.42	abc
DMF	Jalisco	0.98	±	0.04	bcdef	0.75	±	0.03	f	0.08	±	0.01	a	1.07	±	0.02	f	5.20	±	0.14	a
DMF	Veracruz	1.01	±	0.01	bcdef	0.72	±	0.01	f	0.08	±	0.00	a	0.99	±	0.00	f	4.95	±	0.07	a
DMF	Chiapas	0.94	±	0.07	def	0.79	±	0.05	ef	0.09	±	0.02	a	1.10	±	0.07	f	4.45	±	0.35	abc
High producing hybrids and varieties		0.98	±	0.19	A	1.25	±	0.11	B	0.12	±	0.05	AB	1.80	±	0.13	B	2.00	±	0.70	C
Landraces		1.07	±	0.41	A	1.39	±	0.52	A	0.13	±	0.06	AB	1.78	±	0.67	B	2.41	±	1.03	BC
Hybrids mixtures		1.09	±	0.14	A	1.48	±	0.11	A	0.14	±	0.07	A	2.10	±	0.21	A	2.84	±	1.05	B
Dry masa flours		0.98	±	0.02	A	0.80	±	0.08	C	0.08	±	0.01	B	1.13	±	0.11	C	4.61	±	0.39	A

H = Hybrid maize; V = Maize varieties; L = Landraces; M = Hybrid mixtures; DMF = Dry masa flours. Means with a different letter(s) within genotypes and groups of genotypes columns are statistically different (*p* < 0.05). All data was reported as mean ± standard deviation. BLD: Below limit of detection.

Landraces and dry masa flours had a higher and lower RVA Setback, respectively (Figures 1B,D). Larger values in RVA setback indicates more regions integrated into the amylopectin helixes within the crystalline lamellae (5). Higher setback and breakdown viscosities indicated a higher staling on tortillas (25). Also, the higher the percentage of starch, the less pregelatinized starchy masa samples were obtained [C3 (ρ = 0.633), Peak 1 (ρ = 0.600)]. The range of total starch (%) in the samples was between 72.3 to 81.4% (Table 5). The time–temperature profile used during lime cooking and steeping is aimed to achieve partial gelatinization of the starch. When moisture and heat penetrate the endosperm, the starch granules begin to swell water, increasing their size and volume. The masa samples from dry masa flours presented the least retrogradation (Table 4; Figure 1), yielding softer tortillas (Table 6). On the other hand, the masa samples from landraces were the ones that presented the greatest retrogradation (Table 4). It was found that the least pregelatinized masa samples were those produced with landraces and hybrid mixtures (Figures 1A,B), while the masa samples with more pregelatinized starch were produced with dry masa flours (Table 4). Relevant advantage of dry masa flour is that it can be easily blended with hydrocolloids among other additives (20) giving them more desirable characteristics. There was no statistical difference between sample groups in pasting temperature (Figure 1), contradicting previous publications, where it was observed that grains of high-oil maize variety had higher gelatinization temperature than landraces (19). The viscoelastic properties of masa (RVA), the consistency characteristics by Mixolab, as well as the texture of tortillas (strength and extensibility), and rollability on different days also correlated with each other (Supplementary Table S4).

**Table 5:** Analysis of proximate composition and total phenolic content of tortilla samples (dry base).

Sample		Protein				Ash				Dietary fiber ƚ			Fat				Carbohydrates ƚ			Total Starch (%)				Total phenolics (mg/100 g)							
																								Free				Bound			
H	Corteva P4279W	8.15	±	0.02	e	1.48	±	0.07	abcd	8.50	±	1.87	3.14	±	0.26	b	78.73			73.26	±	0.96	fgh	20.5	±	3.03	ij	87.97	±	5.92	ij
H	Corteva P4028W	9.25	±	0.00	abcde	1.37	±	0.13	bcdefg	8.10	±	1.78	2.95	±	0.21	b	78.33			72.32	±	0.46	h	18.3	±	2.16	j	78.51	±	2.15	j
L	Olotillo	10.46	±	0.22	abcd	1.41	±	0.07	bcdef	10.20	±	2.25	4.09	±	0.01	ab	73.84			78.51	±	0.87	b	32.1	±	4.48	efghi	137.84	±	9.23	efghi
L	Serrano Mixe	9.87	±	0.28	abc	1.22	±	0.00	cdefg	10.00	±	2.21	3.37	±	0.34	b	75.54			75.01	±	0.25	efg	32.6	±	3.44	efghi	126.23	±	8.85	fghij
L	Chalqueño	10.14	±	0.49	a	1.03	±	0.05	g	10.90	±	2.40	2.80	±	0.10	b	75.13			73.58	±	0.45	fgh	31.0	±	1.46	efghij	132.93	±	6.25	efghij
H	Bayer DEKALB 2037	8.36	±	0.22	e	1.15	±	0.04	efg	11.30	±	2.50	3.73	±	0.06	b	75.46			73.26	±	0.36	fgh	42.0	±	4.83	bcde	180.43	±	20.78	bcdef
L	Native Texhuaca	11.28	±	0.10	a	1.28	±	0.21	cdefg	9.00	±	1.96	2.75	±	0.14	b	75.69			74.29	±	0.96	efgh	29.2	±	3.95	fghij	125.22	±	8.42	fghij
H	Bayer Antilope/Berrendo	9.78	±	0.27	abcde	1.42	±	0.10	bcdefg	9.30	±	2.05	2.25	±	0.11	b	77.25			74.19	±	0.38	efgh	30.7	±	4.19	efghij	131.83	±	6.88	fghij
L	Native Blue	9.69	±	0.41	abcde	1.55	±	0.05	abcd	9.60	±	2.10	3.13	±	0.13	b	76.03			76.25	±	0.18	cde	30.1	±	3.92	efghij	108.54	±	5.79	ghij
H	Bayer DEKALB 4050	8.95	±	0.13	cde	1.38	±	0.04	bcdefg	6.80	±	1.50	2.10	±	0.04	b	80.77			74.33	±	0.81	efgh	21.0	±	1.34	hij	90.11	±	5.77	ij
V	INIFAP Quality Protein Maize	9.40	±	0.16	abcde	1.42	±	0.24	bcdef	11.30	±	2.49	3.15	±	0.08	b	74.73			74.78	±	0.85	efg	33.0	±	0.25	efghi	188.63	±	1.41	bcde
V	INIFAP High oil corn	10.59	±	0.03	ab	1.38	±	0.01	bcdefg	10.10	±	2.22	5.78	±	0.67	a	72.15			73.34	±	0.97	fgh	33.3	±	4.28	efgh	143.1	±	18.39	efghi
M	Nuevo León	8.45	±	0.25	de	1.47	±	0.06	abcdef	7.20	±	1.57	2.67	±	0.20	b	80.21			75.23	±	0.85	def	24.8	±	2.59	fghij	106.36	±	11.12	ghij
M	Estado de México	9.17	±	0.41	bcde	1.27	±	0.04	cdefg	8.80	±	1.95	3.22	±	0.48	b	77.54			74.34	±	0.32	efgh	24.9	±	2.19	fghij	106.66	±	9.38	ghij
M	Bajío	8.78	±	0.28	bcde	1.19	±	0.02	defg	11.90	±	2.63	2.40	±	1.07	b	75.73			77.36	±	0.49	bcd	24.1	±	3.32	ghij	103.24	±	12.83	hij
M	Jalisco	9.30	±	0.35	abcde	1.16	±	0.07	fg	11.20	±	2.47	2.93	±	0.47	b	75.41			74.54	±	0.84	efg	24.5	±	0.38	fghij	105.05	±	1.63	ghij
M	Veracruz	8.91	±	0.30	cde	1.32	±	0.12	cdefg	10.00	±	2.21	3.04	±	0.68	b	76.73			81.43	±	0.96	a	25.3	±	1.96	fghij	108.57	±	8.39	ghij
M	Chiapas	8.58	±	0.56	e	1.55	±	0.17	abc	9.40	±	2.07	2.86	±	0.46	b	77.61			77.67	±	0.95	bc	34.6	±	5.17	defg	148.42	±	60.79	defgh
DMF	Nuevo León	9.04	±	0.30	bcde	1.69	±	0.11	ab	9.60	±	2.12	2.90	±	0.25	b	76.77			74.44	±	0.57	efgh	28.0	±	3.76	fghij	120.23	±	20.44	ghij
DMF	Estado de México	9.16	±	0.35	bcde	1.80	±	0.06	a	9.60	±	2.12	3.85	±	0.36	ab	75.59			73.19	±	0.95	fgh	62.9	±	9.25	a	269.97	±	39.71	a
DMF	Bajío	8.98	±	0.34	bcde	1.82	±	0.07	a	10.00	±	2.21	4.05	±	0.40	ab	75.15			74.62	±	0.51	efg	46.6	±	2.87	bcd	199.83	±	12.33	bcd
DMF	Jalisco	9.41	±	0.33	abcde	1.43	±	0.02	bcdef	9.80	±	2.15	3.79	±	0.69	ab	75.57			74.15	±	0.32	efgh	37.1	±	5.25	cdef	159.17	±	6.84	cdefg
DMF	Veracruz	8.58	±	0.13	cde	1.50	±	0.05	abcde	10.70	±	2.35	2.91	±	0.03	b	76.31			72.91	±	0.37	gh	52.0	±	7.92	ab	223.19	±	3.98	ab
DMF	Chiapas	8.78	±	0.29	e	1.56	±	0.08	abc	10.30	±	2.28	3.38	±	0.41	b	75.98			73.47	±	0.77	fgh	47.9	±	0.13	bc	205.82	±	0.54	bc
High producing hybrids and varieties		9.21	±	0.77	B	1.37	±	0.10	B	9.34	±	1.56	3.30	±	1.14	A	76.78	±	2.67	73.64	±	0.77	B	28.4	±	8.08	B	128.65	±	41.67	B
Landraces		10.29	±	3.87	A	1.30	±	0.51	B	9.94	±	3.75	3.23	±	1.28	A	75.25	±	28.05	75.53	±	28.19	AB	31.0	±	11.60	B	126.15	±	47.88	B
Hybrids mixtures		8.87	±	0.33	B	1.33	±	0.16	B	9.75	±	1.69	2.85	±	0.29	A	77.21	±	1.73	76.76	±	2.69	A	26.3	±	4.06	B	113.05	±	17.42	B
Dry masa flours		8.99	±	0.26	B	1.63	±	0.15	A	10.00	±	0.40	3.48	±	0.45	A	75.90	±	0.53	73.80	±	0.64	B	45.8	±	11.02	A	196.37	±	47.29	A

H = Hybrid maize; V = Maize varieties; L = Landraces; M = Hybrid mixtures; DMF = Dry masa flours. Means with a different letter(s) within genotypes and groups of genotypes columns are statistically different (*p* < 0.05). ƚ Sample withouth significant difference between samples or between groups. All data was reported as mean ± standard deviation.

**Table 6:** Characteristics of tortilla quality: texture.

Sample		Average breaking force (N)																Average extensibility strength (mm)															
		Day 0				Day 1				Day 3				Day 7				Day 0				Day 1				Day 3			Day 7				
H	Corteva P4279W	11.2	±	2.1	a	9.4	±	1.6	a	8.7	±	1.8	a	7.1	±	0.8	a	12.0	±	0.8	a	9.7	±	0.7	a	9.7	±	1.4	ab	7.0	±	0.5	bc
H	Corteva P4028W	7.3	±	0.8	b	8.1	±	1.4	ab	8.3	±	1.3	ab	5.4	±	0.3	ab	10.2	±	1.7	abc	7.9	±	0.3	abcdef	8.7	±	1.2	abc	6.7	±	0.7	bc
L	Olotillo	4.4	±	0.4	defghij	4.1	±	0.7	efgh	4.4	±	0.7	efghi	3.9	±	0.4	bcdefg	8.9	±	0.8	bcd	6.5	±	0.6	cdefg	6.8	±	1.2	cdef	6.0	±	0.2	c
L	Serrano Mixe	3.8	±	0.8	fghij	3.6	±	0.7	fgh	2.7	±	0.3	i	3.8	±	0.7	bcdefg	8.7	±	1.0	bcd	5.7	±	0.6	g	4.6	±	0.4	f	5.6	±	0.6	c
L	Chalqueño	3.4	±	0.5	hij	2.6	±	0.4	h	3.4	±	0.6	ghi	3.4	±	0.7	cdefg	8.2	±	0.8	cd	5.9	±	0.5	efg	10.5	±	1.0	a	6.1	±	0.6	c
H	Bayer DEKALB 2037	4.3	±	0.5	defghij	3.6	±	0.2	fgh	3.8	±	0.5	efghi	4.3	±	0.7	bcdefg	9.7	±	0.7	abcd	6.2	±	0.4	fg	5.8	±	1.1	def	5.7	±	0.2	c
L	Native Texhuaca	3.1	±	0.4	hij	3.9	±	0.5	fgh	3.3	±	0.4	ghi	4.2	±	0.8	bcdefg	8.4	±	1.1	cd	6.7	±	0.9	cdefg	6.6	±	0.9	def	6.2	±	1.0	c
H	Bayer Antilope/Berrendo	4.0	±	0.7	fghij	5.1	±	0.9	def	4.5	±	0.4	efghi	4.4	±	0.6	bcdefg	9.6	±	1.1	abcd	9.2	±	0.9	ab	7.9	±	0.6	bcd	7.2	±	1.2	bc
L	Native Blue	6.3	±	0.8	bcde	7.3	±	0.7	bc	4.8	±	0.7	defghi	4.8	±	0.7	bc	9.3	±	0.9	bcd	7.5	±	0.6	bcdefg	6.7	±	0.8	cdef	6.4	±	0.3	c
H	Bayer DEKALB 4050	6.7	±	0.5	bcd	5.7	±	0.6	cde	5.7	±	0.6	cdef	3.4	±	0.3	cdefg	10.2	±	0.3	abcd	7.9	±	0.4	abcd	7.0	±	0.3	cde	6.1	±	1.5	c
V	INIFAP Quality Protein Maize	6.7	±	1.2	bc	6.7	±	0.2	bcd	5.0	±	0.9	defgh	4.7	±	0.5	bcd	9.4	±	0.9	abcd	8.0	±	0.5	abcdef	6.9	±	0.6	cdef	11.1	±	1.9	a
V	INIFAP High oil corn	3.2	±	0.6	hij	3.5	±	0.7	fgh	3.9	±	0.8	efghi	3.1	±	0.3	defg	7.7	±	0.2	d	6.0	±	0.4	defg	5.9	±	0.3	def	6.3	±	1.2	c
M	Nuevo León	3.8	±	0.6	fghij	3.7	±	0.3	fgh	6.1	±	1.0	bcde	5.0	±	0.9	bc	9.3	±	0.7	bcd	8.2	±	1.3	abc	7.2	±	1.3	cde	7.2	±	0.9	bc
M	Estado de México	5.6	±	0.9	bcdefg	4.4	±	0.7	efgh	5.2	±	0.3	defg	4.5	±	0.7	bcde	8.9	±	1.3	bcd	6.3	±	0.6	defg	6.5	±	0.3	def	9.3	±	1.3	ab
M	Bajío	6.4	±	1.2	bcde	4.8	±	0.6	defg	7.4	±	1.0	abc	7.0	±	0.9	a	9.5	±	0.6	abcd	7.6	±	0.7	bcdef	7.5	±	0.3	cde	7.7	±	0.4	bc
M	Jalisco	7.3	±	1.3	b	5.7	±	0.7	cde	6.7	±	1.2	bcd	7.0	±	1.0	a	10.1	±	0.8	abc	7.5	±	0.6	bcdef	7.3	±	1.0	cde	7.3	±	1.2	bc
M	Veracruz	5.7	±	0.9	bcdef	5.1	±	0.8	def	4.5	±	0.4	efghi	4.6	±	0.6	bcd	10.0	±	1.4	abc	6.9	±	0.7	cdefg	6.4	±	0.5	def	6.2	±	0.4	c
M	Chiapas	5.1	±	0.9	cdefgh	5.0	±	0.9	def	4.3	±	0.6	efghi	4.4	±	0.9	bcde	10.3	±	0.8	abc	7.1	±	0.4	cdefg	6.4	±	0.6	def	6.8	±	1.1	c
DMF	Nuevo León	3.2	±	0.4	fghij	3.2	±	0.5	gh	2.8	±	0.4	i	3.4	±	0.6	cdefg	11.4	±	0.3	ab	8.1	±	0.3	abc	6.1	±	0.4	def	6.0	±	0.3	c
DMF	Estado de México	3.0	±	0.5	ij	3.3	±	0.6	gh	3.2	±	0.5	hi	3.3	±	0.4	cdefg	9.5	±	1.0	abcd	6.6	±	0.9	cdefg	5.6	±	0.2	ef	6.1	±	0.2	c
DMF	Bajío	2.9	±	0.3	hij	3.2	±	0.6	gh	2.7	±	0.3	i	3.0	±	0.5	efg	11.4	±	1.1	ab	7.0	±	0.8	cdefg	6.1	±	0.6	def	6.1	±	0.6	c
DMF	Jalisco	3.0	±	0.4	ghij	2.6	±	0.3	h	3.8	±	0.7	fghi	2.7	±	0.4	g	10.8	±	0.6	abc	7.0	±	0.9	cdefg	6.7	±	0.2	cdef	6.1	±	1.3	c
DMF	Veracruz	3.0	±	0.1	ghij	2.8	±	0.5	h	2.8	±	0.6	i	2.6	±	0.5	fg	10.9	±	0.0	abc	7.0	±	0.6	cdefg	6.5	±	1.1	def	6.4	±	0.6	c
DMF	Chiapas	2.3	±	0.3	j	2.9	±	0.4	h	3.1	±	0.4	hi	2.9	±	0.2	efg	9.4	±	0.7	bcd	6.9	±	1.1	cdefg	6.2	±	1.1	def	5.8	±	0.5	c
High producing hybrids and varieties		6.2	±	2.50	A	6.0	±	2.05	A	5.7	±	1.88	A	4.6	±	1.23	B	9.9	±	1.17	A	7.8	±	1.28	A	7.4	±	1.32	A	7.2	±	1.69	A
Landraces		4.2	±	1.87	B	4.3	±	2.15	B	3.7	±	1.56	B	4.0	±	1.56	B	8.7	±	3.26	B	6.5	±	2.48	B	7.1	±	3.15	AB	6.0	±	2.26	B
Hybrids mixtures		5.6	±	1.19	A	4.8	±	0.70	B	5.7	±	1.25	A	5.4	±	1.25	A	9.7	±	0.55	A	7.3	±	0.66	A	6.9	±	0.49	AB	7.4	±	1.04	A
Dry masa flours		2.9	±	0.28	C	3.0	±	0.22	C	3.1	±	0.37	B	3.0	±	0.29	C	10.6	±	0.82	A	7.1	±	0.46	AB	6.2	±	0.38	B	6.1	±	0.17	B

H = Hybrid maize; V = Maize varieties; L = Landraces; M = Hybrid mixtures; DMF = Dry masa flours. Means with a different letter(s) within genotypes and groups of genotypes columns are statistically different (*p* < 0.05). ƚ Sample without significant difference between samples or between groups. All data was reported as mean ± standard deviation.

The rate and degree of swelling depend on the organization of starch-protein matrices, which are different in the vitreous and floury endosperm (10, 26). A negative correlation was found between the amount of carbohydrates and protein (Table 5) in tortillas (ρ = −0.577). These morphological differences could promote incomplete/partial starch gelatinization, chemical degradation (oxidation) of starch granules, surface damage that allows lime to penetrate inside the granules and react with amylose and amylopectin molecules, decreasing their size and molecular weight. Therefore, these general differences affected the final functional properties of tortillas (24). Structural composition of grains affects thermal and rheological properties and, consequently, the texture of tortillas (5).

### 3.4. Tortilla quality assessment

In the thermal analysis of samples of nixtamalized tortillas using a differential scanning calorimeter (DSC), the endotherms obtained showed the fusion of retrograde amylopectin. The nixtamalization process of tortillas causes that more than 70% of the starch is gelatinized (27). Therefore, the starch gelatinization endotherm characteristic was not observed in the tortilla thermograms. Instead, an endotherm was observed at lower temperatures between 44 and 69°C, which is like what was previously reported (27, 28). This endotherm behavior is attributed to the fusion of retrograded amylopectin. Also, in increased enthalpies for starch retrogradation, the amylose-lipid complexes did not present a pattern correlated with staling (25). Sample groups did not show significant differences in the onset temperature of melting of recrystallized amylopectin (To), Peak temperature of melting of recrystallized amylopectin (Tp), Final temperature of melting of recrystallized amylopectin (Tf), or in the enthalpy (Supplementary Table S2). Chemical composition had been previously correlated well with kernel shape, starch thermal (enthalpy) and rheological properties (5).

On the other hand, the nixtamalization temperature and the alkaline conditions denatured the proteins and, in combination with the changes of the starch granules, promoted some of their unique rheological characteristics (29, 30). At higher protein concentration (Table 5), lower extensibility of tortillas was observed in texture tests on day 0 (ρ = −0.734; Table 6). Interestingly, tortillas produced with QPM variety had extensibility values at day 7 similar to those of freshly made tortillas with other genotypes (Table 6). Extensibility was related to the ability to roll up and flexibility of the tortilla. Lower extensibilities were found in aged tortillas compared to their fresh counterparts and lower extensibility (12.34%) in tortillas produced with landraces compared to HPHV (Table 6).

Protein content negatively affected the breaking force parameter in texture and rollability. The breaking force is correlated with the hardness of the tortillas detected in the mouth and their resistance to manual tearing. Spherical probe puncturing measures the resistance of the tortilla to being cut by teeth or torn by hand in a better way than flat tip punch or tension. The maximum puncture force with a spherical probe is a useful parameter, as it provides information on the toughness of the tortilla and its ability to support a filling, such as those used to make “tacos.” Changes were observed in the breaking strength of the stored tortillas as they became hard and brittle. After storage, tortillas made from HPHV and landraces did not statistically show any difference in terms of breaking strength, but they did with those produced with dry masa flours, which after 7 days were softer (Table 6).

The greatest difference in rollability occurred on day 7 of tortilla storage (Table 7), where the tortillas produced from landrace kernels showed a significant difference against the other groups (HPHV, hybrid mixture and dry masa flours), presenting lower (−34.4%) rollability values. The landrace samples averaged 1.27 percentage points more protein than other samples (Table 5). It has been documented that protein affected tortilla elasticity, firmness, and smoothness (5). Tortillas have recently been supplemented to increase their protein content with jumbo squid muscle and cricket protein hydrolysates showing tending to become harder to roll without cracking as storage elapsed and resulting in tortillas with low hardness and extensibility values, respectively, (31, 32).

**Table 7:** Rollability of tortillas evaluated through a week old.

Sample		Rollability														
		Day 0 ƚ			Day 1				Day 3				Day 7			
H	Corteva P4279W	4.3	±	0.6	3.3	±	0.5	bc	3.0	±	0.8	abc	3.5	±	1.0	ab
H	Corteva P4028W	4.3	±	0.6	3.8	±	0.5	ab	4.0	±	0.8	a	4.0	±	1.0	ab
L	Olotillo	5.0	±	0.0	3.8	±	1.0	ab	3.5	±	0.6	ab	2.5	±	0.6	abcd
L	Serrano Mixe	4.8	±	0.5	3.8	±	0.5	ab	1.8	±	0.5	c	1.0	±	0.0	d
L	Chalqueño	4.8	±	0.5	4.3	±	0.5	ab	2.0	±	0.0	bc	2.0	±	0.8	bcd
H	Bayer DEKALB 2037	5.0	±	0.0	4.0	±	0.0	ab	2.8	±	1.0	abc	3.3	±	1.0	abc
L	Native Texhuaca	5.0	±	0.0	2.3	±	0.5	c	2.8	±	0.5	abc	1.5	±	0.6	cd
H	Bayer Antilope/Berrendo	5.0	±	0.0	3.8	±	0.5	ab	3.8	±	0.5	a	3.8	±	0.5	ab
L	Native Blue	4.8	±	0.5	5.0	±	0.0	a	3.8	±	1.0	a	3.5	±	0.6	ab
H	Bayer DEKALB 4050	5.0	±	0.0	4.0	±	0.8	ab	4.0	±	0.0	a	3.5	±	0.5	ab
V	INIFAP Quality Protein Maize	5.0	±	0.0	4.3	±	0.5	ab	3.8	±	0.5	a	3.3	±	1.0	abc
V	INIFAP High oil corn	5.0	±	0.0	3.5	±	0.6	bc	2.0	±	0.0	bc	2.0	±	0.0	bcd
M	Nuevo León	5.0	±	0.0	3.5	±	0.6	bc	3.0	±	0.8	abc	3.3	±	1.0	abc
M	Estado de México	5.0	±	0.0	4.0	±	0.0	ab	3.5	±	0.6	ab	3.3	±	0.5	abc
M	Bajío	5.0	±	0.0	4.0	±	0.8	ab	3.5	±	0.6	ab	3.5	±	0.6	ab
M	Jalisco	5.0	±	0.0	3.8	±	0.5	ab	4.0	±	0.0	a	3.0	±	1.6	abc
M	Veracruz	5.0	±	0.0	4.0	±	0.0	ab	3.8	±	0.5	a	3.5	±	0.6	ab
M	Chiapas	5.0	±	0.0	4.0	±	0.0	ab	3.5	±	0.6	ab	3.0	±	0.0	abc
DMF	Nuevo León	5.0	±	0.0	4.0	±	0.0	ab	3.3	±	0.5	abc	3.0	±	0.0	abc
DMF	Estado de México	4.8	±	0.5	4.0	±	0.0	ab	3.0	±	0.0	abc	2.5	±	1.0	abcd
DMF	Bajío	5.0	±	0.0	4.0	±	0.0	ab	3.5	±	0.6	ab	2.3	±	0.5	abcd
DMF	Jalisco	4.8	±	0.5	3.5	±	0.6	bc	3.5	±	0.6	ab	2.8	±	0.5	abcd
DMF	Veracruz	4.5	±	0.6	4.0	±	0.0	ab	3.8	±	0.5	a	3.8	±	0.5	ab
DMF	Chiapas	4.8	±	0.5	4.0	±	0.0	ab	4.0	±	0.0	a	4.0	±	0.0	a
High producing hybrids and varieties		4.8	±	0.30	3.8	±	0.31	A	3.3	±	0.70	AB	3.3	±	0.64	A
Landraces		4.9	±	1.81	3.8	±	1.64	A	2.8	±	1.25	B	2.1	±	1.11	B
Hybrids mixtures		5.0	±	0.00	3.9	±	0.21	A	3.5	±	0.33	A	3.3	±	0.22	A
Dry masa flours		4.8	±	0.17	3.9	±	0.19	A	3.5	±	0.32	A	3.0	±	0.64	A

H = Hybrid maize; V = Maize varieties; L = Landraces; M = Hybrid mixtures; DMF = Dry masa flours. Means with a different letter(s) within genotypes and groups of genotypes columns are statistically different (*p* < 0.05). ƚ Sample without significant difference between samples or between groups. All data was reported as mean ± standard deviation.

The rollability of the tortillas correlated with the amount of protein (ρ = −0.531) and carbohydrates (ρ = 0.579) in the tortilla, as well as pericarp (ρ = −0.626) and optimal grain cooking time (ρ = −0.886); as textural properties of tortillas are affected by the type of endosperm (5).

Total phenolic compounds had a significant effect on texture (at least a correlation was observed) and their interaction with starch affected viscoelastic properties and masa consistency (Supplementary Table S4.). The concentration of free total phenolic content affected the functional behavior of starch as it interacts with starch crystals (33). Phenolic compounds are associated with the cell wall by its interaction with arabinoxylans, other polysaccharides, and proteins (34, 35). The degraded, rich in arabinoxylans, pericarp, acts as a hydrocolloid imparting desirable textural properties to tortillas (20, 36). The concentration of phenolic compounds (Table 5) resulted in tortillas with less rollability, extensibility, hardness and greater masa pregelatinization, and less retrogradation (Supplementary Table S4). In addition, the concentration of phenolic compounds affected the physicochemical properties of starch, increasing the solubility index in water, decreasing the viscosity and hardness, adhesiveness, cohesion and elasticity of the products (33). Therefore, their release during lime-cooking can be an excellent indicator of cell wall degradation, starch damage, and functional properties of nixtamalized products. Furthermore, sample preparation and analysis might be easier to replicate using spectrophotometric methods.

Sensory evaluation tests indicated that panelists generally rated with better acceptability the tortillas made from dry masa flours (Table 8) but it was not statistically significant. The interactions with other macromolecules of the grain types could have affected the quality properties of the tortillas as fat and carbohydrate have been previously negatively correlated with aroma, whereas protein have been positively correlated (5). The landrace native blue, had a lot of variation among the panelists which made it not statistically different. Blare et al. (37) reported that blue corn tortillas were better evaluated when compared to white tortillas and that consumers are willingness to pay up to 42% for tortillas of this genotype.

**Table 8:** Color and sensory analysis of 1 day old tortillas.

Sample		Color												Sensory analyses																	
		L*				a*				b*				Color				Odor ƚ			Flavor ƚ			Texture				Overall acceptability			
H	Corteva P4279W	73.28	±	0.99	fg	0.04	±	0.40	bcd	22.45	±	0.97	bc	7.0	±	1.6	a	7.3	±	1.1	6.9	±	1.7	6.6	±	2.0	abcde	6.6	±	1.5	abcd
H	Corteva P4028W	74.75	±	1.32	efg	0.20	±	0.37	b	21.87	±	1.12	cd	6.9	±	1.7	a	7.4	±	0.8	6.7	±	0.9	6.5	±	1.4	abcde	6.7	±	1.2	abcd
L	Olotillo	75.34	±	1.17	defg	−0.53	±	0.12	bcde	18.37	±	0.89	efgh	6.7	±	1.1	a	7.2	±	1.0	6.2	±	1.2	6.8	±	1.5	abcd	7.0	±	0.8	abc
L	Serrano Mixe	65.79	±	1.13	h	6.14	±	0.72	a	36.12	±	1.92	a	6.4	±	2.1	a	6.7	±	1.6	6.3	±	1.3	6.0	±	2.2	bcde	6.3	±	1.6	bcd
L	Chalqueño	74.44	±	1.90	efg	−0.83	±	0.23	de	17.92	±	1.68	efghi	7.1	±	0.7	a	6.8	±	0.9	6.5	±	1.6	5.3	±	2.0	e	6.1	±	1.5	bcd
H	Bayer DEKALB 2037	76.23	±	2.93	def	−0.79	±	0.13	de	17.54	±	2.23	fghi	6.3	±	1.4	a	6.9	±	1.5	6.6	±	1.3	5.7	±	2.2	de	6.9	±	1.4	abc
L	Native Texhuaca	72.74	±	1.72	fg	0.17	±	0.55	bc	21.91	±	1.49	cd	7.2	±	1.1	a	6.8	±	1.8	6.6	±	1.3	6.9	±	1.2	abcd	6.6	±	1.4	abcd
H	Bayer Antilope/Berrendo	77.61	±	2.07	cde	−0.40	±	0.83	bcde	17.55	±	1.66	fghi	7.0	±	1.3	a	6.7	±	1.3	6.5	±	1.4	5.7	±	2.1	de	6.2	±	1.5	bcd
L	Native Blue	44.68	±	2.34	i	−1.96	±	0.30	f	0.43	±	1.27	j	7.4	±	1.6	a	6.7	±	1.3	6.4	±	1.5	6.8	±	1.2	abcd	6.7	±	1.3	abcd
H	Bayer DEKALB 4050	75.53	±	1.78	defg	−0.42	±	0.23	bcde	19.29	±	1.08	cdefg	6.4	±	1.5	a	6.4	±	1.3	5.4	±	1.8	5.7	±	1.3	de	5.6	±	1.2	d
V	INIFAP Quality Protein Maize	72.40	±	1.39	g	−0.72	±	0.26	cde	20.68	±	1.21	cdef	7.3	±	1.3	a	7.2	±	1.1	6.6	±	1.2	6.6	±	1.5	abcde	6.7	±	1.0	abcd
V	INIFAP High oil corn	74.53	±	1.36	efg	−1.12	±	0.16	ef	21.00	±	1.40	cde	6.6	±	1.7	a	6.4	±	2.0	6.9	±	1.3	6.8	±	1.5	abcd	6.9	±	1.2	abc
M	Nuevo León	74.83	±	1.34	efg	−0.35	±	0.53	bcde	25.46	±	1.82	b	7.2	±	1.6	a	6.8	±	1.4	7.0	±	1.3	7.9	±	0.9	a	7.2	±	1.1	ab
M	Estado de México	74.43	±	1.11	efg	−0.65	±	0.23	bcde	19.43	±	1.38	cdefg	6.7	±	1.6	a	6.3	±	1.2	6.0	±	1.9	5.8	±	1.9	cde	6.2	±	1.5	bcd
M	Bajío	73.03	±	1.25	fg	−0.35	±	0.18	bcde	19.00	±	0.94	defg	6.6	±	1.0	a	6.6	±	1.2	5.8	±	1.1	5.9	±	1.7	cde	5.9	±	1.2	cd
M	Jalisco	73.60	±	1.97	fg	−0.44	±	0.25	bcde	18.64	±	1.83	efg	6.6	±	1.1	a	6.6	±	1.4	6.5	±	1.1	5.9	±	1.6	cde	6.1	±	1.4	bcd
M	Veracruz	73.90	±	1.18	fg	−0.35	±	0.19	bcde	18.77	±	1.28	defg	6.2	±	1.5	a	6.1	±	1.3	6.3	±	1.5	5.3	±	1.5	e	5.7	±	1.6	d
M	Chiapas	74.09	±	0.85	efg	0.03	±	0.14	bcd	20.24	±	0.74	cdef	6.4	±	0.8	a	5.5	±	1.0	6.1	±	1.1	5.8	±	1.5	cde	6.2	±	1.0	bcd
DMF	Nuevo León	82.75	±	0.94	a	−0.10	±	0.32	bcd	16.28	±	0.29	ghi	7.2	±	1.1	a	6.7	±	1.5	6.2	±	1.4	7.2	±	1.2	abc	6.6	±	1.3	ab
DMF	Estado de México	82.91	±	1.04	a	−0.70	±	0.17	cde	15.17	±	1.03	i	7.0	±	1.0	a	6.2	±	1.9	6.2	±	1.5	6.7	±	1.5	abcde	6.9	±	1.4	abc
DMF	Bajío	82.70	±	0.99	a	−0.67	±	0.28	bcde	15.38	±	0.69	hi	7.2	±	1.7	a	5.8	±	1.9	6.2	±	1.2	5.7	±	1.6	de	6.1	±	1.5	bcd
DMF	Jalisco	82.30	±	0.48	ab	−0.41	±	0.19	bcde	16.35	±	0.94	ghi	7.3	±	1.0	a	7.7	±	0.9	7.7	±	1.0	7.4	±	1.3	ab	7.6	±	1.2	a
DMF	Veracruz	81.14	±	0.59	abc	−0.15	±	0.33	bcd	16.45	±	0.54	ghi	7.3	±	0.9	a	7.6	±	0.7	7.4	±	0.7	7.6	±	1.4	a	7.6	±	1.0	a
DMF	Chiapas	78.82	±	1.40	bcd	0.14	±	0.67	bc	19.70	±	1.89	cdef	7.6	±	0.5	a	6.8	±	1.0	7.2	±	1.2	6.9	±	1.7	abcd	7.2	±	1.0	ab
High producing hybrids and varieties		74.90	±	1.63	B	−0.46	±	0.43	B	20.05	±	1.83	A	6.8	±	0.35	AB	6.9	±	0.4	6.5	±	0.5	6.2	±	0.47	AB	6.5	±	0.43	AB
Landraces		66.60	±	26.93	C	0.60	±	2.62	A	18.95	±	12.56	A	7.0	±	2.61	AB	6.8	±	2.6	6.4	±	2.4	6.4	±	2.43	AB	6.5	±	2.45	AB
Hybrids mixtures		73.98	±	0.63	B	−0.35	±	0.22	AB	20.26	±	2.62	A	6.6	±	0.34	B	6.3	±	0.5	6.3	±	0.4	6.1	±	0.91	B	6.2	±	0.53	B
Dry masa flours		81.77	±	1.44	A	−0.31	±	0.30	AB	16.55	±	1.49	A	7.3	±	0.17	A	6.8	±	0.7	6.8	±	0.6	6.9	±	0.63	A	7.0	±	0.53	A

H = Hybrid maize; V = Maize varieties; L = Landraces; M = Hybrid mixtures; DMF = Dry masa flours. Means with a different letter(s) within genotypes and groups of genotypes columns are statistically different (*p* < 0.05). ƚ Sample without significant difference between samples or between groups. All data was reported as mean ± standard deviation.

Tortillas produced from landraces and dry masa flours were darker and lighter in color, respectively (Table 8). Color is the result of cob and kernel color, concentration of lime during nixtamalization, extent of nixtamal washing, and the final pH. Tortillas produced with dry masa flour had lower pH (Table 2), generally, less alkaline pHs yield lighter colorations (22). It is important to note that the quality of the tortillas was not only affected by the maize genotype used. Starch can also be affected by phenotypic factors such as temperature, available water, place of growth, environmental stress, climatic variations, soil fertility, atmospheric composition, among others, and sometimes these factors have a greater influence on starch biosynthesis than the genotype (38).

## 4. Conclusion

The physical and chemical properties of the different types of maize kernels affected processability and the quality of tortillas. It is important to emphasize that no difference was found among high producing hybrids and varieties (HPHV), landraces, hybrid mixtures and dry masa flours in 21 evaluated parameters. The HPHV, especially Corteva and Bayer genotypes, showed better properties in all processing stages, and the proximal composition of the landraces showed slightly higher protein values, therefore, more research is needed to investigate the nutritional effects of hybrids versus landraces (e.g. protein digestibility, amino acid profile). It should be remembered that hybrids were developed from landraces to improve yields and in this breeding process some characteristics were lost.

Apparently, there is not a perfect maize genotype, but there are sources or raw materials to make mixtures according to the objective and end use of DMF. An ideal maize is one that is highly productive in the field, processes well industrially and yields a good quality final product.

## Data availability statement

The raw data supporting the conclusions of this article will be made available by the authors, without undue reservation.

## Author contributions

BA-E, SS-S, and CC-H contributed to conception and design of the study. BA-E and CC-H organized the database and performed the statistical analysis. BA-E wrote the first draft of the manuscript. CC-H wrote sections of the manuscript. SS-S revised it critically for important intellectual content. All authors contributed to the article and approved the submitted version.

## Conflict of interest

The authors declare that the research was conducted in the absence of any commercial or financial relationships that could be construed as a potential conflict of interest.

## Publisher’s note

All claims expressed in this article are solely those of the authors and do not necessarily represent those of their affiliated organizations, or those of the publisher, the editors and the reviewers. Any product that may be evaluated in this article, or claim that may be made by its manufacturer, is not guaranteed or endorsed by the publisher.

## Supplementary material

The Supplementary material for this article can be found online at:

https://www.frontiersin.org/articles/10.3389/fnut.2023.1105619/full#supplementary-material
