# Supplementary material for: Quality assessment of maize tortillas produced from landraces and high yield hybrids and varieties
Source: Front Nutr. 2023 Feb 9;10:1105619. doi: 10.3389/fnut.2023.1105619 (PMC9948077; doi:10.3389/fnut.2023.1105619)
Supplement: Supplementary file 1 [file Table_1.pdf]

Supplementary Table 1. Masa particle size distribution

	Sample	Masa particle size distribution*			
		d(0.1) µm	d(0.5) µm	d(0.9) µm	d(0.98) µm
H	Corteva P4279W	14.54	61.19	258.47	428.86
H	Corteva P4028W	14.07	57.85	270.88	441.09
L	Olotillo	13.00	63.18	287.68	451.36
L	Serrano Mixe	13.85	69.83	287.01	450.13
L	Chalqueño	10.31	51.64	275.8	443.2
H	Bayer DEKALB 2037	12.75	57.21	252.91	425.87
L	Native Texhuaca	10.02	54.58	273.14	438.02
H	Bayer Antilope/Berrendo	13.50	67.57	271.84	437.71
L	Native Blue	9.65	38.19	251.78	426.51
H	Bayer DEKALB 4050	11.18	44.99	274.29	441.82
V	INIFAP Quality Protein Maize	11.01	60.52	286.81	447.04
V	INIFAP High oil corn	12.05	73.52	299.23	457.5
M	Nuevo León	13.77	64.08	260.65	429.55
M	Estado de México	11.31	58.17	281.02	442.8
M	Bajío	10.89	50.02	284.09	448.96
M	Jalisco	10.55	47.14	281.57	447.52
M	Veracruz	11.20	43.31	265.08	435.07
M	Chiapas	11.08	43.53	268.53	439.38
DMF	Nuevo León	19.46	145.51	362.66	494.81
DMF	Teotihuacán	17.94	138.6	357.9	492.15
DMF	Bajío	23.97	147.65	357.54	491.41
DMF	Jalisco	20.25	152.06	369.01	498.48
DMF	Chinameca	29.82	170.9	380.25	504.94
DMF	Chiapas	30.85	151.41	360.77	493.36
	High producing hybrids and varieties	12.73 ± 1.38 B	60.41 ± 8.93 B	273.49 ± 15.81 B	439.98 ± 10.72 B
	Landraces	11.37 ± 1.92 B	55.48 ± 12.04 B	275.08 ± 14.56 B	441.84 ± 10.13 B
	Hybrids mixtures	11.47 ± 1.16 B	51.04 ± 8.41 B	273.49 ± 9.95 B	440.55 ± 7.44 B
	Dry masa flours	23.72 ± 5.51 A	151.02 ± 10.89 A	364.69 ± 8.69 A	495.86 ± 5.10 A

H = Hybrid maize; V= Maize varieties; L = Landraces; M= Hybrid mixtures; DMF =Dry masa flours. Means with a different letter(s) within groups are statistically different ($p < 0.05$).

*d(0.1), d(0.5), d(0.9), d(0.98), maximum diameter of 10%, 50% and 90% of the particles. Average results of 3 replicates with coefficient variation < 5%
