# Supplementary material for: Quality assessment of maize tortillas produced from landraces and high yield hybrids and varieties
Source: Front Nutr. 2023 Feb 9;10:1105619. doi: 10.3389/fnut.2023.1105619 (PMC9948077; doi:10.3389/fnut.2023.1105619)
Supplement: Supplementary file 2 [file Table_2.pdf]

Supplementary Table 2. Differential Scanning Calorimetry (DSC) Analysis*

	Sample	To (°C)	Tp (°C)	Tf (°C)	ΔH (J/g)
H	Corteva P4279W	45.73	54.51	62.36	4.39
H	Corteva P4028W	60.06	66.45	68.69	0.09
L	Olotillo	48.1	48.79	59.97	2.98
L	Serrano Mixe	48.26	56.12	62.5	4.22
L	Chalqueño	46.97	56.63	59.01	4.35
H	Bayer DEKALB 2037	48.86	52.95	59.07	2.81
L	Native Texhuaca	45.41	53.62	57.82	4.23
H	Bayer Antilope/Berrendo	46.78	54.46	62.55	5.86
L	Native Blue	47.8	54.45	61.48	6.51
H	Bayer DEKALB 4050	44.59	53.61	61.67	6.00
V	INIFAP Quality Protein Maize	44.99	52.76	59.44	5.54
V	INIFAP High oil corn	47.92	55.09	62.29	5.48
M	Nuevo León	51.7	52.95	56.58	10.74
M	Estado de México	44.73	52.6	59.58	4.64
M	Bajío	45.33	53.95	62.59	7.22
M	Jalisco	44.24	54.45	64.95	5.57
M	Veracruz	46.85	53.44	59.38	2.66
M	Chiapas	45.86	53.1	59.41	5.18
DMF	Nuevo León	47.89	55.23	61.61	6.39
DMF	Teotihuacán	52.04	55.71	61.64	4.39
DMF	Bajío	48.23	55.35	61.49	3.23
DMF	Jalisco	47.04	55.68	62.92	5.32
DMF	Chinameca	48.65	55.17	60.06	4.80
DMF	Chiapas	48.26	55.66	61.61	6.59
	High producing hybrids and varieties	48.42 ± 5.36 a	55.69 ± 4.82 a	62.30 ± 3.16 a	4.31 ± 2.17 a
	Landraces	47.31 ± 1.17 a	53.92 ± 3.12 a	60.16 ± 1.87 a	4.46 ± 1.28 a
	Hybrids mixtures	46.45 ± 2.73 a	53.42 ± 0.68 a	60.42 ± 2.93 a	6.00 ± 2.75 a
	Dry masa flours	48.69 ± 1.73 a	55.47 ± 0.24 a	61.56 ± 0.91 a	5.12 ± 1.27 a

*Retrograde amylopectin temperatures - To: Onset temperature of melting of recrystallized amylopectin, Tp: Peak
H = Hybrid maize; V= Maize varieties; L = Landraces; M= Hybrid mixtures; DMF =Dry masa flours. Means with a different letter(s) within groups are statistically different ($p < 0.05$).
Average results of 3 replicates with coefficient variation < 5%
