# Supplementary material for: Quality assessment of maize tortillas produced from landraces and high yield hybrids and varieties
Source: Front Nutr. 2023 Feb 9;10:1105619. doi: 10.3389/fnut.2023.1105619 (PMC9948077; doi:10.3389/fnut.2023.1105619)
Supplement: Supplementary file 3 [file Table_3.pdf]

Supplementary Table 3. Yields in the tortilla making process

		Yield (%)			
	Sample	Kernel to Nixtamal	Nixtamal to Masa	Masa to Tortilla	Flour to masa
H	Corteva P4279W	164.30	109.78	70.44	-
H	Corteva P4028W	164.29	114.67	74.38	-
L	Olotillo	180.67	103.11	73.44	-
L	Serrano Mixe	173.00	110.67	78.66	-
L	Chalqueño	183.33	106.22	74.28	-
H	Bayer DEKALB 2037	178.67	110.44	76.78	-
L	Native Texhuaca	168.00	112.11	75.00	-
H	Bayer Antilope/Berrendo	178.67	110.00	72.32	-
L	Native Blue	176.33	110.44	76.36	-
H	Bayer DEKALB 4050	168.00	113.78	73.16	-
V	INIFAP Quality Protein Maize	174.67	118.44	79.01	-
V	INIFAP High oil corn	169.00	116.44	79.13	-
M	Nuevo León	168.67	111.78	77.20	-
M	Estado de México	168.67	112.00	78.10	-
M	Bajío	173.00	109.78	79.92	-
M	Jalisco	172.67	112.22	86.05	-
M	Veracruz	165.00	116.67	83.45	-
M	Chiapas	167.33	113.89	80.86	-
DMF	Nuevo León	-	-	81.36	234
DMF	Teotihuacán	-	-	74.21	220
DMF	Bajío	-	-	74.80	233
DMF	Jalisco	-	-	73.23	236
DMF	Chinameca	-	-	72.07	235
DMF	Chiapas	-	-	75.12	228
	High producing hybrids and varieties	171.08 ± 6.24 A	113.37 ± 3.41 A	75.03 ± 3.37 B	-
	Landraces	176.27 ± 6.09 A	108.51 ± 3.73 B	75.55 ± 2.04 B	-
	Hybrids mixtures	169.22 ± 3.10 A	112.72 ± 2.33 AB	80.93 ± 3.33 A	-
	Dry masa flours	-	-	75.13 ± 3.25 B	231 ± 6.07

H = Hybrid maize; V= Maize varieties; L = Landraces; M= Hybrid mixtures; DMF =Dry masa flours. Means with a different
Average results of 3 replicates with coefficient variation < 5%
