# Supplementary material for: Quality assessment of maize tortillas produced from landraces and high yield hybrids and varieties
Source: Front Nutr. 2023 Feb 9;10:1105619. doi: 10.3389/fnut.2023.1105619 (PMC9948077; doi:10.3389/fnut.2023.1105619)
Supplement: Supplementary file 4 [file Table_4.pdf]

Supplementary Table 4. Pearson correlation of the masa and tortilla quality parameters

Variable 1	Variable 2	ρ
Free phenolics	Breaking Force day 3 (Texture)	-0.797
Free phenolics	Rollability day 3	-0.791
Free phenolics	Breaking Force day 1 (Texture)	-0.767
Free phenolics	Breaking Force day 7 (Texture)	-0.744
Total phenolics	Breaking Force day 0 (Texture)	-0.74
Tmax gelatinization (DSC)	Rollability day 0	-0.694
Breaking Force day 1 (Texture)	Peak time (RVA)	-0.689
Minimum torque (Mixolab)	Setback (RVA)	-0.679
Free total phenolics	Breaking Force day 1 (Texture)	-0.674
Breaking Force day 0 (Texture)	Peak time (RVA)	-0.67
Breaking Force day 1 (Texture)	Development time (Mixolab)	-0.665
Breaking Force day 1 (Texture)	Stability time (Mixolab)	-0.655
Free phenolics	Extensibility strength day 1 (Texture)	-0.58
Free phenolics	Setback (RVA)	-0.524
Free phenolics	Retrogradation (Mixolab)	-0.515
Total phenolics	Starch pregelatinization (Mixolab)	-0.506
Free phenolics	Stability time (Mixolab)	0.508
Free phenolics	Development time (Mixolab)	0.535
Free phenolics	Peak time (RVA)	0.538
Breaking Force day 0 (Texture)	Retrogradation (Mixolab)	0.651
Retrogradation (Mixolab)	Breakdown (RVA)	0.652
Breaking Force day 0 (Texture)	Setback (RVA)	0.655
Gelatinization degree (Mixolab)	Breakdown (RVA)	0.673
Gelatinization degree (Mixolab)	Peak 1 (RVA)	0.675
Gelatinization degree (Mixolab)	Final Viscocity (RVA)	0.711
Development time (Mixolab)	Peak time (RVA)	0.729
